# The effect of calcium phosphate nanoparticles on hormone production and apoptosis in human granulosa cells

**DOI:** 10.1186/1477-7827-8-32

**Published:** 2010-04-02

**Authors:** Xiaohui Liu, Dingxia Qin, Yugui Cui, Liang Chen, Hao Li, Zhen Chen, Li Gao, Ying Li, Jiayin Liu

**Affiliations:** 1 Center of Clinical Reproductive Medicine, First Affiliated Hospital, Jiangsu Key Laboratory of Reproductive Medicine, Nanjing Medical University, Nanjing 210029, China; 2 Department of Mechanical and Aerospace Engineering, University of Missouri, Columbia, MO 65211, USA; 3 Department of Civil & Environmental Engineering, University of Missouri, Columbia, MO 65211, USA; 4 Jiangsu Family Planning Institute, Nanjing 210036, China; 5 Suzhou Municipal Hospital & Suzhou Medical Center for Maternal and Child Health, Suzhou 215002, China

## Abstract

**Objectives:**

Although many nanomaterials are being used in academia, industry and daily life, there is little understanding about the effects of nanoparticles on the reproductive health of vertebral animals, including human beings. An experimental study was therefore performed here to explore the effect of calcium phosphate nanoparticles on both steroid hormone production and apoptosis in human ovarian granulosa cells.

**Methods:**

Calcium phosphate nanoparticles uptaking was evaluated by transmission electron microscopy (TEM). The cell cycle was assessed with propidium iodide-stained cells (distribution of cells in G0/G1, S, and G2/M phases) by flow cytometry. The pattern of cell death (necrosis and apoptosis) was analyzed by flow cytometry with annexin V-FITC/PI staining. The expression of mRNAs encoding P450scc, P450arom and StAR were determined by RT-PCR. Progesterone and estradiol levels were measured by radioimmunoassay.

**Results:**

TEM results confirmed that calcium phosphate nanoparticles could enter into granulosa cells, and distributed in the membranate compartments, including lysosome and mitochondria and intracellular vesicles. The increased percentage of cells in S phase when cultured with nanoparticles indicated that there was an arrest at the checkpoint from phase S-to-G2/M (from 6.28 +/- 1.55% to 11.18 +/- 1.73%, p < 0.05). The increased ratio of S/(G2/M) implied the inhibition of DNA synthesis and/or impairment in the transition of the S progression stage. The apoptosis rate of normal granulosa cells was 7.83 +/- 2.67%, the apoptotic rate increased to 16.53 +/- 5.56% (P < 0.05) after the cells were treated with 100 microM calcium phosphate nanoparticles for 48 hours. Treatment with calcium phosphate nanoparticles at concentrations of 10-100 microM didn't significantly change either the progesterone or estradiol levels in culture fluid, and the expression levels of mRNAs encoding P450scc, P450arom and StAR after 48 h and 72 h period of treatment.

**Conclusion:**

Calcium phosphate nanoparticles interfered with cell cycle of cultured human ovarian granulosa cells thus increasing cell apoptosis. This pilot study suggested that effects of nanoparticles on ovarian function should be extensively investigated.

## Background

Nanoparticles possess nanostructure-dependent properties due to their small size, chemical composition, surface charge, solubility and/or shape [1]. Despite the wide applications of nanomaterials, there is a serious lack of information concerning the impact of manufactured nanomaterials on human health and the environment. Typically, the nanoparticles are small enough to penetrate through very small capillaries into the human tissues and cells. Because nanoparticles can pass through biological membranes, they can affect the physiology of most cells, including brain and testes [2-4]. Application of nanomaterials leads to considerable concern regarding its potential biological effects and toxicity in humans [5,6]. The major toxicological concern is that some of the manufactured nanomaterials are redox active [7,8], and some particles transport across cell membranes, especially into the mitochondria [9]. The reported findings were controversial.

Hydroxyapatite (HA, a kind of calcium phosphate) nanoparticles are similar to human bone in chemical composition and have long been appreciated for their biocompatibility. HA is now one of the most widely used materials in the bone-repairing field. Calcium phosphate is the primary mineral phase of human and animal bone and tooth. Such a mineral phase, with its plate-like or needle-like shape, typically varies in size from a very few to hundreds of nanometers. HA nanomaterial has also been studied for various applications, including orthopedics, dentistry, and food science, with many research topics involving the mineral's interaction with cells [10].

Reproduction is a complex biological process that is particularly sensitive to environmental endocrine disruptors. Many chemicals have negative impacts on gametogenesis and hormone reproduction by either directly affecting germ cells and indirectly affecting on somatic nursing cells [11]. Ovarian granulosa cells play a major role in maintaining ovarian function, health, and female fertility. Cadmium oxide at lower concentrations promoted apoptosis rather than necrosis in the mammalian germline stem cells, thus leaving the plasma membrane intact [12]. Some intracellular organelles involved in steroidogenesis were infiltrated and/or altered due to the presence of the nanogold particles [11]. Calcium phosphate nanoparticles have been used clinically so that we will study carefully their effect on the female reproductive system and reproductive health in the future work.

The hypothesis has been made that HA nanopaticles themselves and many regulatory agents delivered by this kind of nanopaticles could have some effects on steroidgenesis and follicular development and maturation *in vivo *and *in vitro*. In this pilot paper, we firstly investigated the effect of calcium phosphate nanoparticles itself on hormone production and apoptosis in human granulosa cells cultured *in vitro*.

## Methods

### Chemicals

**Figure 1:**
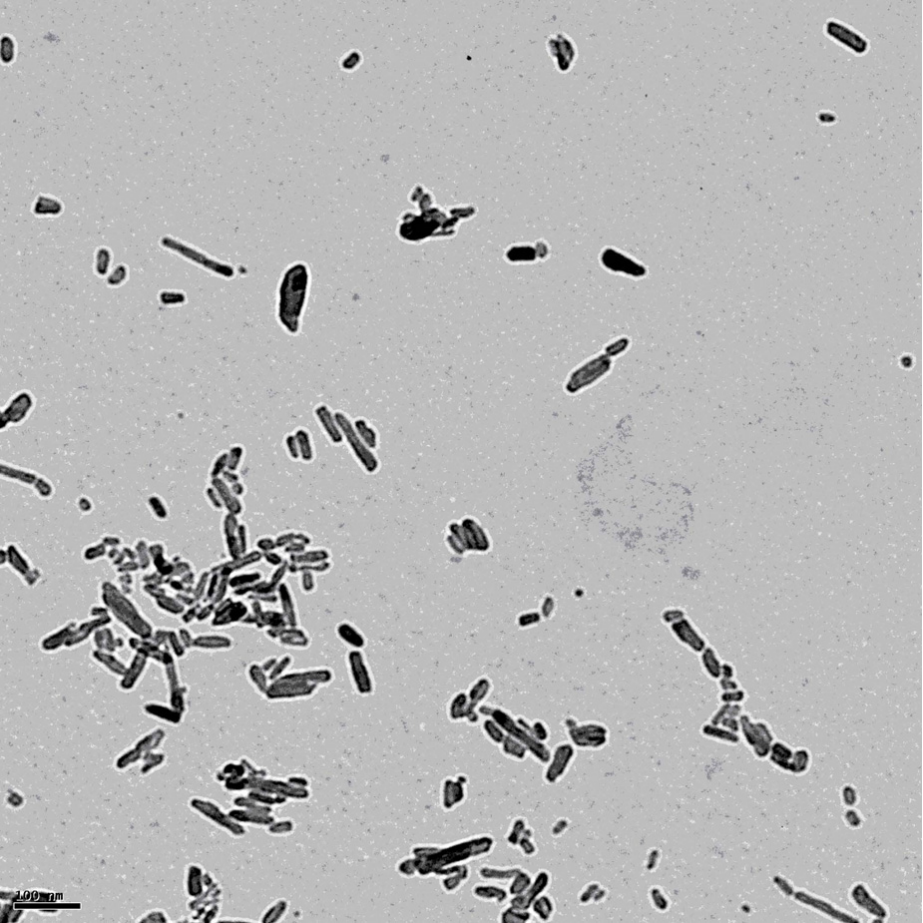
**The morphology of hydroxyapatite nanoparticles**. The nanoparticles were observed by transmission electron microscopy (TEM, JEOL-1400TEM, USA) with an acceleration voltage of 100 kV. The scale bar in the image is 100 nm.

A solution with a Ca/P ratio of 1.67 was prepared with 60 mM of Ca(NO_3_)_2_·4H_2_O and 36 mM of NaH_2_PO_4_·2 H_2_O [10,13,14]. The solution was stirred vigorously and heated to 85°C and then the same volume of concentrated ammonium hydroxide solution (28-30% NH_3_) was quickly added to the solution, which immediately induced nanoparticle precipitation. The mixture was held at 85°C for approximately 24 hours to ensure a complete conversion of the starting material to HA. After fabrication, the mixture was then cooled to room temperature and the liquid part was then diluted until the pH dropped below 9 without any HA nanoparticles being lost. The rod-like crystal was used in this study (Fig 1). The somallest was 20-30 nm, and particles less 10% were greater than 100 nm. Dry particle of HA prepared is stable in room temperature.

4-androstene-3,17-dione, Percoll and fetal bovine serum (FBS) were purchased from Sigma-Aldrich (St. Louis, MO). Collagenase Type I was obtained from Worthington Biochemical Corp. (Lakewood, NJ). Antibiotic-antimycotic preparation (penicillin, 10,000 IU/mL; streptomycin, 10,000 mg/mL; amphotericin B, 25 mg/mL) were purchased from Grand Island Biological Co. (Grand Island, NY). HEPES was purchased from American Bioanalytical (Natick, MA).

### Cell culture

#### Isolation of granulosa cells

Granulosa cells for *in vitro *experimentation were collected from infertilite women who undergoing IVF-ET treatment [11,15]. The women's follicles were punctured with a hypodermic needle to extrude granulosa cells into dish, then, centrifuged (200 × *g*, 5 min) and resuspended in DMEM/F12 containing antibiotics and 10% FBS. The blood cells were separated by 50% Percoll and centrifuged at 600 × *g *for 5 min. Written consent was provided by all patients. The research project was approved by the Human Research Ethics Committee of First Affiliated Hospital, Nanjing Medical University.

### Cell culture

Every time, we can get samples from 8-10 patients. Those cells, after separated by Percoll, were pooled together, in which the inter-individual variability should be eliminated or be a low level. Cells were then cultured in DMEM/F12 supplemented with l-glutamine (2 mL), 10% FBS, penicillin (10,000 IU/mL), streptomycin (10,000 mg/mL) and amphotericin B (25 mg/mL) at 37°C in an atmosphere of 5% CO_2_. Cultures were carried out in 6-well plates (Corning Glass Works, Corning, NY) and 12-well plates. The cultured cells in the 12-well plates were used for hormone analysis and those in the 6-well plates were used for isolation of mRNAs and cell cycle analysis.

### HA nanoparticles treatment

After an initial 72 h of culture in DMEM/F12 medium with 10% FBS, the culture medium was carefully removed and replaced with fresh medium. Next, the granulosa cells were simultaneously exposed to different concentrations of calcium phosphate nanoparticles. In addition, 4-androstene-3,17-dione (final concentration, 500 nM) was added into the granulosa cell medium as a substrate for estrogen biosynthesis. Ethanol was added to the negative control cells to exclude the effect of the dissolvent. All experiments were duplicated 3 or 4 times. The experiment of every batch was grouped as follows. Group I was treated with only 4-androstene-3,17-dione as control; Group II was treated with HA nanoparticles (10 μM) and 4-androstene-3, 17-dione; Group III was treated with HA nanoparticles (100 μM) and 4-androstene-3,17-dione. The culture medium in the 12-well plates was collected after 48 h and stored at -80°C for hormone analysis. Cells cultured in the 6-well plates were lysed with TRIzol and stored at -80°C for mRNAs analysis. For each experiment each assay was performed in triplicate. Cells cultured in the 6-well plates were also fixed in 80% ethanol for cell cycle analysis.

### Cell preparation for TEM

Granulosa cells were exposed during 2 h, 4 h, 24 h, 48 h and 72 h to nanoparticles (100 μM). They were fixed with 2.5% glutaraldehyde, post-fixed with OsO_4 _and dehydrated in graded concentrations of ethanol [8,9] then embedded in Epon. Ultra-thin sections were cut (80 nm), counterstained with lead citrate and uranyl acetate then observed with a Philips CM 12 electron microscope at 80 kV.

### Cell cycle analysis

5 × 10^5 ^granulosa cells were plated in 6-well plates and incubated in DMEM/F12 with 0.5% FBS for 48 h to synchronize the cells in G0/G1 phase by serum deprivation [16,17]. Cells were then induced to re-enter the cell cycle by incubation in fresh medium with the calcium phosphate nanoparticles at the final concentration (determined from the growth assay results) for a further 48 h.

The cellular DNA content was determined by flow cytometry, as described previously. We collected the floating and attached cells using pancreatic enzyme and resuspended them in DMEM/F12. The cells were fixed for 30 min in an ice-cold 80% ethanol solution containing ribonuclease (RNase, 2 mg/ml). We washed the cells in PBS, and then stained them with propidium iodide (PI) for 10 min. The PI-elicited fluorescence was measured for individual cells using a flow cytometer (FACSCalibur, Becton, Dickinson & Co., Tokyo, Japan) with laser excitation at 488 nm. Emissions greater than 590 nm were collected in a linear/log scale fashion. We analyzed a total of 1 × 10^6 ^cells for each sample and determined the percentages of cells in G0/G1, S, and G2/M phases using standard ModiFIT and CELLQUEST software (Becton, Dickinson & Co.).

### Radioimmunoassay

The concentrations of progesterone and estradiol in medium were assessed using commercially available radioimmunoassay kits (Bei Fang Biotechnology Corp., Beijing, China) according to the manufacturer's protocol. The intra-assay and inter-assay variations of the kits were both less than 10%.

### Semi-quantitative RT-PCR

After the supernatants were collected for steroid hormone analysis, the cells in the corresponding wells were harvested for mRNA extraction using Trizol reagent according to the manufacturer's protocol. Extracted RNA was treated with DNase prior to measuring RNA content by spectrometry at OD260/280. Equal amounts of RNA (500 ng) were reverse-transcribed into cDNA as previously described [18], using the following thermocycler protocol: 70°C for 5 min and 42°C for 90 min.

The expression of P450scc, P450arom and StAR mRNAs in the granulosa cells were measured by the semi-quantitative RT-PCR. The primer pairs are shown as follows: 1) P450scc: F: 5'-GTGACAATGGCTGGCTAA-3', R: 3'-GAGGAATCGTTCTGGGTT-5', 2) P450arom: F: 5'-CACCTCTAACACGCTCTT-3', R: 3'-AATCAACTCAGTGG CAAA-5, 3) StAR: F: 5'-AGGAAGGACGAAGAACCA-3', R: 3'-CAGCCGAG AACCGAGTAG-5'. PCR was performed in a total volume of 20 μl. β-Actin mRNA abundance served as an internal control [18-21].

### Statistical analysis

Data were expressed as mean ± SD from at least three independent experiments. Statistical analysis was performed using one-way analysis of variance (ANOVA) and *t *test. Values were determined to be significant when *P *< 0.05.

## Results

### Effect of HA nanoparticles on granulosa cells

**Figure 2:**
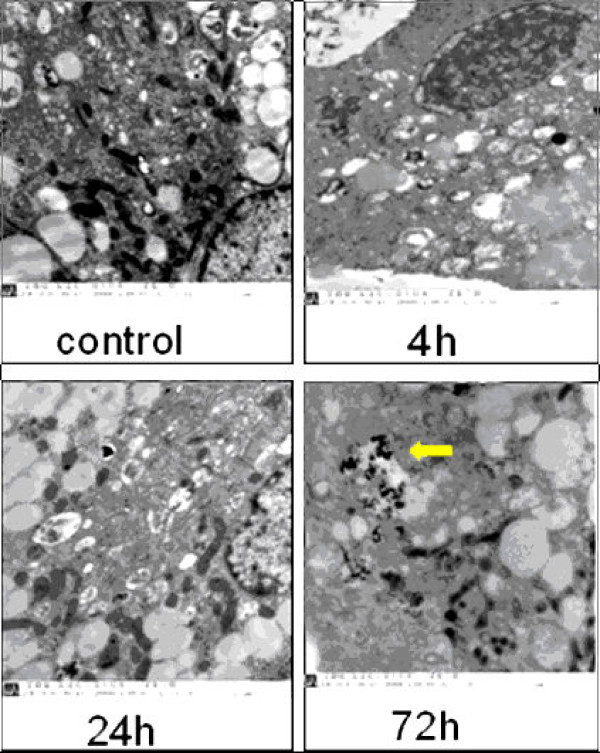
**TEM observation of granulosa cells exposed to calcium phosphate nanoparticles**. Cells were exposed during 72 h to 100 μM of nanoparticles. Nanoparticles were internalized in cells and localized in cytoplasms, mostly in membranate compartments, including lysosome and mitochondria, thecal organelles (arrow). The scale bar in the image is 500 nm.

TEM examination was performed on granulosa cells post 2 h, 4 h, 24 h, 48 h and 72 h exposure to HA nanoparticles. HA nanoparticles were observed in granulosa cells (Fig. 2). Further analysis indicated that HA nanoparticles were localized in the cytoplasm, mostly in membranate compartments, including lysosome and mitochondria, thecal organelles (Fig 2, arrow), besides particles existing out of cells, surrounding cells. Compared with control cells, no visible morphological changes were detected in cells exposed to HA nanoparticles.

### Flow cytometric analysis of cell cycle distribution and apoptosis

**Figure 3:**
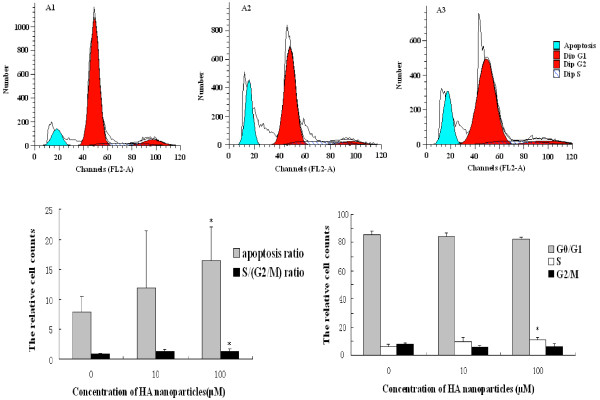
**Apoptotic population and flow cytometric analysis of granulosa cell cycle distribution**. (A) Flow cytometric analysis of calcium phosphate nanoparticles induced apoptosis in granulosa cells using annexin V-FITC/PI. The x-axis represents fluorescent intensity on a logarithmic scale, whereas the y-axis represents the number of events: (A1) Control; (A2) Treatment with 10 μM calcium phosphate nanoparticles; (A3) Treatment with 100 μM calcium phosphate nanoparticles; the green peak refers to the apoptotic population. (B C) The results were analyzed by Mod Fit LT 3.0; Data were presented as mean ± SD (n = 3). *p < 0.05, compared with the control group (0 μM).

**Figure 4:**
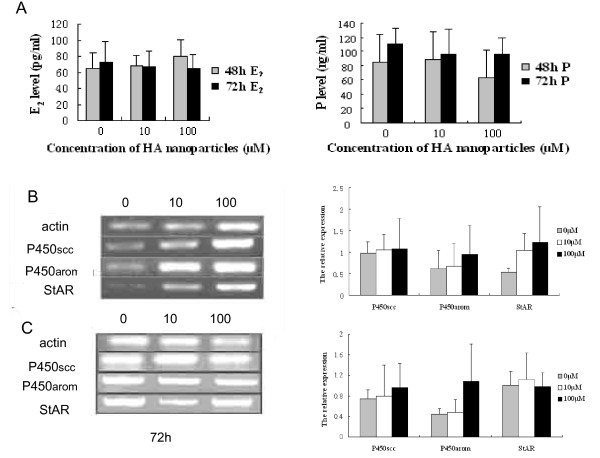
**Effect of calcium phosphate nanoparticles on P450scc, P450arom and StAR mRNA expression in human granulosa cells *in vitro***. Granulosa cells were cultured as described in the text. Following the measurement of steroid hormone levels (A). RT- PCR was used to determine the expression of P450scc, P450arom and StAR mRNAs in the cultured granulosa cells after 48 h (B) and 72 h (C) with calcium phosphate nanoparticles. Experiments were repeated three times during separate times and Agarose gel electrophoresis of the PCR products.

The cell cycle distribution was analyzed by MultiCycle software to investigate the effect of calcium phosphate nanoparticles on the cell cycle, as shown in Fig 3. Granulosa cells were treated with 10 and 100 μM concentrations of calcium phosphate nanoparticles for 48 h. An accumulation of cells in the S phases (from 6.28 ± 1.55% to 11.18 ± 1.73%) was observed. Compared with control, percentage of cells in G2/M phase in Group III increased significantly (p < 0.05) (Fig. 3A). The apoptosis rate of control group was 7.83 ± 2.67%, but the apoptotic population significantly increased to 16.53 ± 5.56% after 48 h treatment with 100 uM calcium phosphate nanoparticles (P < 0.05) (Fig 4A). At the same time, the proportion of the cells in S/(G2/M) phase increased perceptibly (0.89 ± 0.11% to1.38 ± 0.32%) (P < 0.05). The increased percentage of cells in the S phase when treated with HA nanoparticles indicated that there was an arrest at the checkpoint from phase S-to-G2/M, while an increase in the S/(G2/M) ratio implied inhibition of DNA synthesis and/or impairment in the transition or the S progression stage (Fig. 3B).

### Production of progesterone and estradiol and expression of mRNAs encoding P450scc, P450arom and StAR in granulosa cells after treatment with HA nanoparticles

As shown in Fig 4, treatment with calcium phosphate nanoparticles at concentrations of 10 μM and 100 μM (48 h, 72 h) did not cause significant change in either progesterone and estradiol levels (Fig. 4A, *P *> 0.05), and in expression of mRNAs encoding P450scc, P450arom and StAR (Fig 4B, C). By semi-quantitative RT-PCR, we performed a comparative analysis of gene expression in cultured cells exposed to HA nanoparticles and those not exposed (*P *> 0.05).

## Discussion

Despite the potential benefits of nanotechnology, some studies have indicated that certain nanoparticles might cause adverse effects because of their small size and unique properties [22]. For example, HA is now one of the most widely used materials in the bone-repairing field or used as medical adjuvant. Nanomaterials can enter human tissues through several ports via the lungs after inhalation [23], through the digestive system [24], and possibly through the skin [25]. It has been confirmed that nanoparticles could distributed and/or accumulated in reproductive system, ovary and testis [2,3,22]. Due to their small size, chemical composition, surface charge, solubility and/or shape, nanoparticles could affect hormone production and fertility, and reproductive health.

TEM observation suggested that calcium phosphate nanoparticles entered into the cultured human granulosa cells and located in thecal organelles within cytoplasm, including lysosome and mitochondria besides particles existing out of cells, surrounding cells (Fig 2). By our protocol prepared HA nanoparticles, two kinds of hydroxyapatite with different nanocrystal morphology were obtained via a simple aqueous precipitation method under different reactants molar ratios. Under Ca/P molar ratio of 1.67/1, rod-like crystal was produced, while under Ca/P molar ratio of 1.80/1, spherical crystal was produced [13,14,21]. The rod-like crystal was used in this study (Fig 1). Dry HA particle prepared is stable in room temperature, we usually stored at 4°C in the refrigerator for longer time over months. The suspended particles were not stable within medium and within cell. Small particles could polymerizat while large particles could schizolysis, totally, calcium phosphate nanoparticles in medium trended to aggregation. So, the suspension of HA nanoparticles in medium was freshly prepared once before the beginning of the each experiment. Based on others' modeling work, nanoparticles in 20-30 nm are easier to penetrate cell walls and larger size make this more difficult [11,26,27]. We found that HA nanoparticles distributed in membranate compartments within cells (Fig 2). It was also shown by others [13,14,21,28]. There are two kinds of endocytosis, phagocytosis and pinocytosis [29,30] Phagocytosis is restricted to specialized cells such as macrophages, monocytes and neutrophils. Pinocytosis included macropinocytosis (for particles > 1 μm), clathrin-mediated endocytosis (~120 nm), caveolin-mediated endocytosis (~60 nm) or clathrin- and caveolin-independent endocytosis (~90 nm). Due to their size, HA nanoparticles agglomerates were more likely to enter into cells through enter into cells through composite endocytosis.

After treatment with different concentrations of calcium phosphate nanoparticles for 48 h, an accumulation of cells in the S, S/(G2/M) and apoptotic population phases was observed (Fig 3). The cell cycle is a complex process by which cells receive different growth controlling signals that are integrated and processed at various points known as checkpoints [31]. The cell growth is controlled when cells are not allowed to proceed further than these checkpoints. Our data suggested that inhibition of cell growth by HA nanoparticles mainly elicit change in proliferation dynamics, the redistribution of cells along the cell cycle. The increased apoptosis rate was linked to cell cycle arrest. Cadmium oxide nanoparticles at lower concentrations promoted apoptosis rather than necrosis in the mammalian germline stem cells. Mitochondria play a central role in the regulation of apoptotic signaling [32-34]. Potential disord of mitochondria is one of the earliest intracellular events that occur following induction of apoptosis [35]. Thus, calcium phosphate nanoparticles could induce increase of cells arrest in S, S/(G2/M) and increased apoptotic population by interferencing mitochondrial structure and function [32,34] Intracellular free calcium level could also be increased by HA treatment. Pezzatini S et al used HA concentrations ranging from 2 to 10 μg/ml to study microvascular endothelial cell [21]. Qiang Fu et al tried HA with 31.25 to 250 μg/ml in Osteosarcoma U2-OS cell [14]. In our study, granulosa cells were treated with HA nanoparticles at two concentrations of 10 and 100 μM, equally 5 and 50 μg/ml. HA particles was suspensed in DMEM/F12 medium, pH did not changed significantly. So, cell apoptosis was unlikely resulted from the elevated alkaline by HA nanoparticles in culture medium.

We evaluated steroid hormone production and mRNAs expression encoding P450scc, P450arom and StAR in granulosa cells exposed to HA nanoparticles for 48 h and 72 h (Fig 4). HA nanoparticles at 10 and 100 μM did not significantly change progesterone and estradiol production. Even though these results did not show the functional disord as well as its molecular mechanisms in this study, the effect of HA nanoparticles on ovarian function should not be underestimated. Effects of nanoparticles on ovarian function should be extensively investigated in future. Stelzer et al recently reported that the amount of estrogen was significantly increased by treatment with 10 nm nanogold particles (2.85 × 10^10 ^particles/ml) for 1, 3, 5 h, conversely attenuated by treatment for 24 h, with rat ovarian granulosa cells [11]. There were differences in dosage, chemicals, particle size, and cell model between our study and Stelzer's report. And the cell models were different. Human luteinized granulosa cells have been exposed to pharmacological doses of exogenous gonadotropins, being in the process of undergoing differentiation to luteal cells with relatively limited capacity for cell proferation [16]. Because of the absence of granulosa cells from the unstimulated, human normal ovaries, these cells cultured *in vitro *now are useful cell model, to study cell cycle regulators, apoptosis, and related mechanisms involved in human ovary granulosa cell [17].

In conclusion, calcium phosphate nanoparticles interfered with cell cycle of cultured human ovarian granulosa cells thus increasing cell apoptosis. HA nanoparticles could enter into granulosa cells by many ways such as directly penetrating and endocytosis, and were located in thecal organelles, including lysosome and mitochondria. Although hormone production of the cultured granulosa cells was not significantly affected by 10 μM and 100 μM HA nanoparticles within 48 h treatment, this pilot study suggested that effects of nanoparticles on ovarian function should be extensively investigated.

## List of abbreviations

HA: Hydroxyapatite; P450arom: P450aromatase; P450scc: cholesterol side chain cleavage enzyme; StAR: steroidogenic acute regulatory protein; TEM: transmission electron microscopy; UFPs: ultrafine particles.

## Competing interests

The authors declare that they have no competing interests.

## Authors' contributions

XL and DQ carried out cell culture, flow cytometry, RIA and molecular studies. YC and JL designed the project. YC, XL and DQ write the manuscript. LC, HL and ZC did nanoparticles and characterization, and helped to review the manuscript. XL and YC carried out TEM. LG participated in samples' work and worked as lab assistant. YL participated in design and performed the statistical analysis. JL was director of the whole project. All authors read and approved the final manuscript.
